# Editorial: The long and short of mental time travel—self-projection over time-scales large and small

**DOI:** 10.3389/fpsyg.2015.00668

**Published:** 2015-05-20

**Authors:** James M. Broadway, Claire M. Zedelius, Jonathan W. Schooler, Simon Grondin

**Affiliations:** ^1^Psychological and Brain Sciences, University of California Santa BarbaraSanta Barbara, CA, USA; ^2^École de psychologie, Université LavalQuébec, QC, Canada

**Keywords:** time perception, consciousness, mental time travel, oscillatory activity, memory, attention, rhythm perception, internal clock

This Research Topic is about time-experience broadly construed, as it manifests at perceptual and conceptual time-scales (milliseconds-to-seconds vs. longer times, respectively). Authors representing a broad spectrum of psychology and neuroscience have contributed, introducing novel theories, empirical findings, and methodological innovations. In this Editorial we give a thematic overview of the exciting and diverse contents of this Research Topic. (Abbreviations: O, Opinion; H&T, Hypothesis and Theory; OR, Original Research; P, Perspective; MR, Mini-Review; GC, General Commentary; M, Methods).

## Consciousness

Many contributors acknowledged that the experience of time is an irreducible part of conscious experience, strongly related to the experience of *being a self* (Wittmann et al., 2015). Zhou, Pöppel, and Bao (P) integrate different aspects of temporal experience into a unified biologically-grounded framework held together by the concepts of *identity* and *homeostasis*. Berkovich-Ohana and colleagues (H&T) locate representations of minimal self and extended self within a three-dimensional consciousness state-space defined by time, awareness, and emotion. Fingelkurts and Fingelkurts (O) link phenomenology to neural activation patterns, which in turn are constrained by bodily space and the larger context, in order to construct the temporal dimensions of past, future, and present. Martin and colleagues (H&T) consider how disturbances of the minimal self in patients with schizophrenia are possibly related to alterations in temporal processing typically associated with this disorder. In order to better illuminate how creative insight is subjectively experienced, with special attention to its temporal aspects, Cosmelli and colleagues (P) recommend neurophenomenological methodologies, integrating cognitive neuroscience with descriptive, first-person data.

## Multiple-scales

Many contributors addressed how time is represented at multiple scales. Wackermann (O) grounds the experience of time in a knowing, willing subject, exploring how subjective horizons determined by human biological constraints impose various preconditions on the “notion and knowledge of time” across multiple scales. Singularly, Bonato and colleagues (O) propose that temporal cognitions respecting events occurring at vastly different time-scales, such as interval timing vs. mental time travel, are nonetheless represented by a common *spatial* metric (Bonato et al., 2012).

## Mechanisms

Many contributors addressed theoretical mechanisms of timing and time perception (Grondin, 2010; Matthews and Meck, 2014). Block and Grondin (GC) argue that the time perception field is too preoccupied theoretically with internal-clock models and empirically with interval timing. Alternatives to internal-clock models are well-represented in the Research Topic. For example, Marchetti (H&T) discusses time-experience as resulting from a process of *construction* performed by sampling and binding of the contents of working memory by attention. Schweickert and colleagues (OR) similarly emphasize the roles of attention and working memory, introducing to the field a novel *complex span and timing hypothesis*, which predicts that only secondary tasks that are sensitive to individual differences in working memory capacity will interfere with performance on the primary task of time perception in a dual-task setting. Perhaps of some importance for this notion, working memory capacity predicts timing abilities, even when these are tested without dual-task interference (Troche and Rammsayer, 2009; Broadway and Engle, 2011) Gupta (H&T) proposes a new theory explaining sub- and supra-second timing performance by calibration of neural oscillators by sensory, motor, and feedback processes.

## Perceptual time

Experimental and theoretical work on time perception has led to increased understanding of how people keep track of time through multimodal perceptual processes (Grondin, 2001). Mitsudo and colleagues (OR) identify event-related brain potentials specifically associated with equality/inequality judgments of successive time intervals marked by auditory cues. Mioni and colleagues (OR) present evidence in favor of distinct timing mechanisms above and below a threshold of 1300 ms based on effects of context and space-time compatibility. Grondin (O) proposes that cross-modality influences on perceiving time raises questions about the realism of a unitary clock hypothesis. Van Rijn (O) demonstrates how abstract mechanisms underlying interval timing are affected by perceived speed, in a simulated driving task. Schaefer (O) speculates that shared components between movement and musical imagery may offer a window into timing and temporal skills, of relevance to cognition beyond movement or auditory functions. Elliott (OR) reports effects on perception due to specific phase relationships among stimuli in backward masking experiments. Zakay (H&T) looks at time perception in relation to *information processing load*, focusing his analysis on the experience of boredom.

## Conceptual time

Experimental and theoretical work on mental time travel has led to better understanding of how time is represented conceptually (Roberts, 2008; Wittmann, 2013). Ye and Song (OR) report that in contrast to adults, the prospective bias in children seems to be unrelated to current task demands. Roy (O) suggests that so-called “optimistic biases,” causing people to under-estimate how much time a particular task will take, are over-stated in the literature. Scarf and colleagues (MR) argue that if “spoon test” performances of children and great apes were assessed by identical criteria, the evidence suggests the ability for mental time travel can be observed among great apes. Logan (O) advocates similarly for the existence of mental time travel among nonhuman animals, focusing on certain bird species, and proposes new research approaches to further advance this question. Friedman and colleagues (M) describe a new *immersive virtual reality* technique in which individuals can virtually travel back in time to re-live a previous life-or-death moral dilemma, with the opportunity to act differently the second time around. Franklin and colleagues (O) challenge traditional concepts of time with notions of precognition and retro-causality.

## Conclusion

To sum up, the collection of articles in this Research Topic showcases the diverse fecundity of theoretical and empirical developments across a wide spectrum of contemporary research into time-experience broadly construed. It should be acknowledged that the organizing distinction between “perceptual” and “conceptual” time-experience is really only a heuristic; but it has enabled this Research Topic to bring into a single view, a wide panorama of ways to think about and investigate time-experience.

### Conflict of interest statement

The authors declare that the research was conducted in the absence of any commercial or financial relationships that could be construed as a potential conflict of interest.
